# Draft Genome Sequences of *bla*_KPC_-Containing Enterobacter aerogenes, Citrobacter freundii, and Citrobacter koseri Strains

**DOI:** 10.1128/genomeA.00035-18

**Published:** 2018-02-22

**Authors:** Tracy H. Hazen, Roberta T. Mettus, Christi L. McElheny, Sarah L. Bowler, Yohei Doi, David A. Rasko

**Affiliations:** a Institute for Genome Sciences, University of Maryland School of Medicine, Baltimore, Maryland, USA; b Department of Microbiology and Immunology, University of Maryland School of Medicine, Baltimore, Maryland, USA; c Division of Infectious Diseases and Center for Innovative Antimicrobial Therapy, University of Pittsburgh School of Medicine, Pittsburgh, Pennsylvania, USA; d Department of Microbiology, Fujita Health University, Aichi, Japan

## Abstract

We report here the draft genome sequences of four *bla*_KPC_-containing bacteria identified as Klebsiella aerogenes, Citrobacter freundii, and Citrobacter koseri. Additionally, we report the draft genome sequence of a K. aerogenes strain that did not contain a *bla*_KPC_ gene but was isolated from the patient who had the *bla*_KPC-2_-containing K. aerogenes strain.

## GENOME ANNOUNCEMENT

Klebsiella pneumoniae carbapenemase (KPC) was initially described in K. pneumoniae; however, the *bla*_KPC_ gene that encodes KPC has since been identified in diverse bacteria, including other Klebsiella species and in Citrobacter species (1–15). Thus, whole-genome sequencing is an important tool for providing insight into the dissemination of the *bla*_KPC_ genes among diverse species, including those that are not a frequent cause of human illness but may serve as reservoirs for antibiotic resistance genes.

The Citrobacter freundii strains (YDC691 and YDC692-2) described in this report were isolated in 2015 from a sputum sample (YDC691) and a bronchoalveolar lavage fluid sample (YDC692-2) from the same patient, collected a week apart. The Klebsiella aerogenes strains (YDC581 and YDC581-4) and Citrobacter koseri strain YDC582 were isolated in 2012 from either blood (YDC581 and YDC581-4) or Jackson-Pratt drain fluids (YDC582), which were collected on the same day from the same patient. The Klebsiella and Citrobacter strains were tested for their susceptibilities to 21 antibiotics by determining their MICs using the Sensititre Gram-negative breakpoint plates. All of the strains exhibited resistance to ticarcillin-clavulanic acid, piperacillin-tazobactam, aztreonam, ceftazidime, and cefotaxime. Also, all of the strains except for YDC581-4 exhibited resistance to ertapenem, and only strain YDC692-2 exhibited resistance to imipenem and doripenem.

Genomic DNA was extracted from the K. aerogenes and Citrobacter strains using the Sigma GenElute genomic DNA kit (Sigma-Aldrich, St. Louis, MO). The genomes were sequenced using paired-end 500-bp-insert libraries on the Illumina HiSeq 4000. The genomes were assembled using SPAdes version 3.7.1 (16) and were filtered to contain only contigs ≥500 bp in length and with ≥5× coverage. Antibiotic resistance genes were detected in each of the genome assemblies using the Comprehensive Antibiotic Resistance Database (CARD) version 1.1.8 (17).

The draft genome assemblies of the C. freundii strains had sizes of 5.17 to 5.74 Mb and a GC content of 51.6%. The C. koseri strain YDC582 had an assembly size of 4.65 Mb and a GC content of 53.78%. The K. aerogenes genome assemblies had sizes of 5.18 and 5.37 Mb and a GC content of 55%. The genome assemblies of the C. freundii strains all contained the *bla*_KPC-3_ gene, as well as the *bla*_OXA-9_, *bla*_SHV-7_, and *bla*_TEM-1_ genes. The strains isolated from the second patient included K. aerogenes strain YDC581, which contained *bla*_KPC-2_, and K. aerogenes strain YDC581-4, which was isolated from the same patient sample but did not contain any detectable *bla*_KPC_ genes. The second patient also had C. koseri strain YDC582, which was isolated from a sample different from that of strains YDC581 and YDC581-4, and it contained a *bla*_KPC-2_ gene. All three strains from this patient also contained a *bla*_TEM-1_ gene.

In summary, we report the draft genome sequences of four *bla*_KPC_-containing K. aerogenes, C. freundii, and C. koseri strains, as well as one K. aerogenes strain that did not contain a *bla*_KPC_ gene. These genome sequences are significant because they represent two sets of strains that were coisolated from samples from two different patients and provide insight into the transmission of the *bla*_KPC_ gene between multiple species from the same patient.

### Accession number(s).

The draft genome assemblies of C. koseri strain YDC582, K. aerogenes strain YDC581-4, K. aerogenes strain YDC581, C. freundii strain YDC691, and C. freundii strain YDC692-2 have been deposited in GenBank under the accession numbers PGIF00000000, PGIG00000000, PGIH00000000, PGII00000000, and PGIJ00000000, respectively.
